# Prenatal stress selectively enhances locomotor hyperactivity in male mice with embryonic loss of *Fgfr2*

**DOI:** 10.3389/fpsyt.2026.1820942

**Published:** 2026-09-03

**Authors:** Michelle X. Chen, Polona Jager, Abigail Sawyer, Hanna E. Stevens

**Affiliations:** 1 Department of Psychiatry, Carver College of Medicine, Iowa City, IA, United States; 2 Child Study Center, Yale School of Medicine, New Haven, CT, United States; 3 University of Iowa Hawk-IDDRC (Intellectual and Developmental Disabilities Research Center), New Haven, CT, United States

**Keywords:** ADHD, fibroblast growth factor receptor 2 (FGFR2), gene-environment interaction, neurodevelopment, prenatal stress

## Abstract

Attention-deficit/hyperactivity disorder (ADHD) is a developmental psychiatric disorder associated with a complex interplay of genetic and environmental risk factors. In a previous study, we found embryonic dorsal forebrain loss in male mice of fibroblast growth factor receptor 2 (*Fgfr2*), which has a critical role in normal brain development, results in ADHD-relevant phenotypes: increased locomotion and sociability, and impaired working memory postnatally. How such genetic vulnerabilities interact with environmental exposures to translationally model human ADHD risk remains unclear. Here, we performed a follow-up study by pairing the embryonic hGFAP-cre *Fgfr2* conditional knockout (*Fgfr2* cKO) mouse model with prenatal repetitive restraint stress, modeling an environmental factor associated with ADHD risk, to assess adult offspring behaviors, dopamine transporter (DAT) levels, and pilot parvalbumin-positive (PV+) cell densities. Offspring of prenatally stressed, *Fgfr2* cKO male mice showed increased locomotion (80% higher compared to non-stressed, *Fgfr2* cKO animals). However, prenatal stress had no significant effects on impulsivity, working memory, or sociability in *Fgfr2* cKO male mice. Neurobiologically, prenatal stress did not significantly affect DAT levels, and DAT protein levels did not correlate with behavior, suggesting DAT dysregulation is not the mechanism of increased locomotion in this model. Taken together, our findings implicate prenatal stress as a potential contributor to some gene-environment interactions for ADHD risk, supporting its use in translational animal models of childhood psychiatric disorders.

## 1 Introduction

Attention deficit hyperactivity disorder (ADHD) is a developmental psychiatric disorder, characterized by symptoms of inattention, impulsivity, and/or hyperactivity (1–3). The prevalence of ADHD is currently estimated to be 11.4% of U.S. children ever diagnosed (4). Most individuals with ADHD also report experiencing co-occurring disorders, such as anxiety, depression, behavioral conduct difficulties, or learning disabilities (4) which complicate treatment efficacy. As a result, ADHD is a public health concern, underscoring the importance of understanding its etiology to improve prevention and intervention strategies.

As with other brain disorders, ADHD arises from a complex interplay of factors. Twin and adoption studies implicate both genetic and environmental etiological factors (1, 2, 5, 6). There are few single gene risks, and the disorder is thought to be polygenic (6–8), making it challenging to behaviorally replicate ADHD with animal models carrying a susceptibility gene. However, emerging transgenic mouse models displaying ADHD-relevant behavioral phenotypes have provided insight into neurobiological mechanisms even if these target genes do not contribute to polygenic risk. Some models include disruption of fibroblast growth factor (FGF) systems. Mice with disruptions in FGF receptors (FGFR1, 2, and 3) exhibit a smaller cerebral cortex, which has also been observed in patients with ADHD (9, 10). Additionally, male mice with FGF disruptions show ADHD-relevant phenotypes (e.g. changes in locomotion and social interaction behavior). These models suggest FGF signaling, which is required for normal formation of the cerebral cortex as well as involved in development of cortical excitatory and inhibitory neurons (11–14), is relevant to psychiatric illness. We previously studied effects of FGFR2 disruption across development. We found embryonic conditional ablation of the *Fgfr2* gene in radial glial stem cells of the dorsal forebrain [(*Fgfr2* conditional knockout (cKO)] (13, 15), thereby affecting neuronal and glial progeny populations, in male mice leads to behaviors relevant to clinical ADHD presentations. Namely, male mice displayed hyperactivity, working memory deficits, and social impulsivity in adulthood. Interestingly, *Fgfr2* loss in adulthood does not replicate such findings (15). This suggests neurodevelopmental mechanisms in the critical embryonic and early postnatal periods may be involved in ADHD risk.

One well-documented environmental risk during embryonic neurodevelopment is prenatal stress (16–18), which refers to maternal psychological distress during pregnancy and associated physiological responses (19, 20). Its link to psychopathological outcomes could be explained by its ability to alter intrauterine conditions and thus disrupt normative fetal brain development (16, 21, 22). Prenatal stress frequently associates with ADHD symptoms in children (17, 23–33). Animal studies further support links between prenatal stress and ADHD-relevant outcomes in offspring and provide insight into neuronal systems affected by prenatal stress (20, 34–47). However, it is not known to what extent prenatal stress combines with genetic vulnerability to affect deficits (27, 48).

Neurobiologically, dopaminergic (DA) system dysfunction, particularly in the cortex and the mesolimbic structures, is thought to be implicated in ADHD (7, 49, 50) and in basic functions disrupted in ADHD (51–53). For example, *dopamine transporter* (*DAT*) gene variant (10-repeat SLC6A3 allele) associates with ADHD (54), and *Dat* knockout mice are used to model ADHD (55). Rodent studies of other ADHD risks find alterations in DA systems. For example, prenatal stress in rodents altered *Nurr1* and *Pitx3* expression, which are molecules implicated in DA neuron development, as well as DA, tyrosine hydroxylase (TH), and DA2 receptor (D2R) levels in the midbrain, nucleus accumbens and cortical regions (20, 56–64). Though there is some discrepancy in directionality of these changes, the DA system remains critical to ADHD etiology and treatment. Additionally, increasing evidence links dysfunction in cortical excitatory-inhibitory balance with ADHD (65, 66). The inhibitory gamma-aminobutyric acid (GABA)-ergic system is crucial for regulating cognitive and motor functions. Parvalbumin-positive (PV+) interneurons are the largest population of GABAergic inhibitory neurons in the cortex, are decreased in ADHD models (7, 11, 67, 68), and are affected by FGF signaling and prenatal stress independently (11, 69).

Here, we aim to follow-up our previous study (15) by exploring a prenatal gene-environment interaction with relevance to ADHD. We leverage the *Fgfr2* cKO mouse model in combination with prenatal stress to assess male offspring adult locomotor activity, working memory, social intrusiveness, and impulsivity- and anxiety-relevant behaviors. To start to understand neurobiological mechanisms through which prenatal stress and genetic vulnerability may predispose to ADHD, we assess DAT protein levels, a key player in the DAergic system, and perform pilot studies measuring forebrain PV+ cells, a key GABAergic interneuron type.

## 2 Materials and methods

### 2.1 Mice

**Figure 1:**
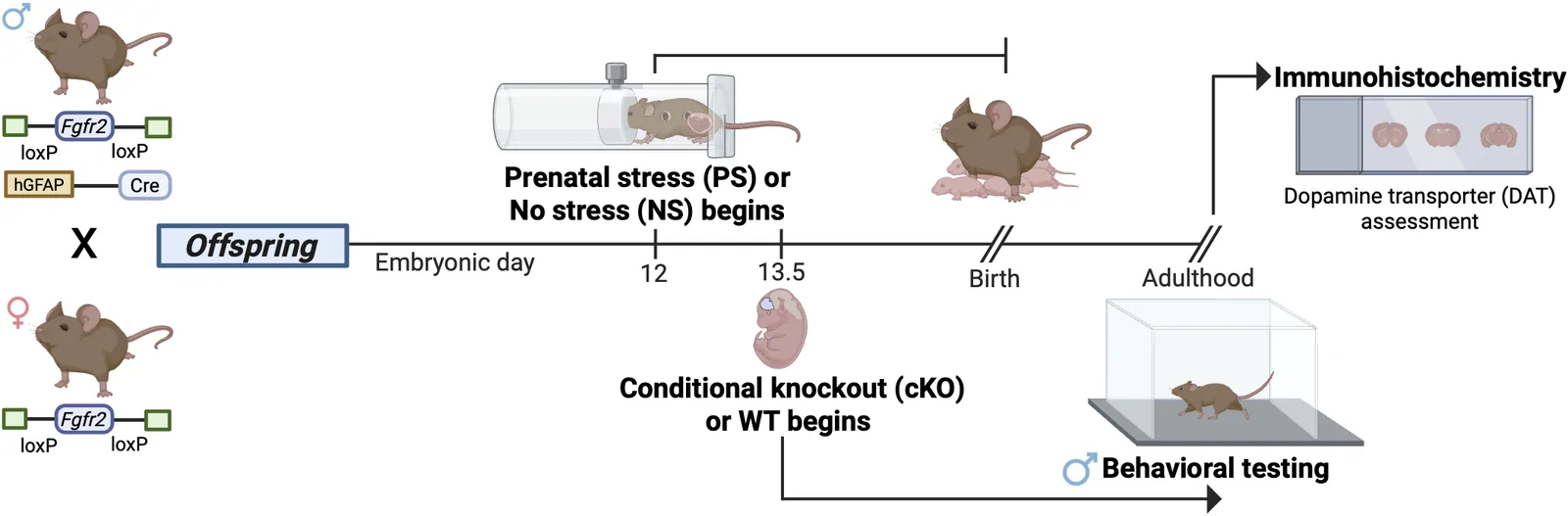
Experimental timeline. Male mice homozygous for Fgfr2f alleles and heterozygous for hGFAP-Cre recombinase were crossed with female mice homozygous for Fgfr2f alleles. Prenatal stress or no-stress conditions began at embryonic day (E)12 until birth. hGFAP expression occurs in all dorsal telencephalic radial glia and astrocytes starting from E13.5. All behavioral testing and neurobiological analyses were performed in adult male mice.

All experimental procedures were approved by the Yale Animal Resources Center and Institutional Animal Care and Use Committee (IACUC) policies in accordance with the National Institutes of Health guidelines. Sufficient animals or samples (n’s for each group noted in each figure caption from 11 non-stressed dams and 8 prenatally-stressed dams) were generated for each assessment using a power analysis based on previous studies and *α* = 0.05 and *β* = 0.2. The experimental design is shown in **Figure 1**.

#### 2.1.1 Prenatal restraint stress

Female mice were checked for vaginal plugs daily to detect embryonic day 0. Mice were randomly assigned to prenatal restraint stress (PS) or non-stress, control conditions (NS). Restraint stress was induced in pregnant mice from E12 onwards until birth (P0). Mice were placed in plexiglass restrainers for 45 minutes, three times daily, under bright lights and during the daytime light cycle (43). Control pregnant mice were continuously housed in normal conditions.

#### 2.1.2 Generation of Fgfr2 cKO animals

Conditional hGFAP-cre *Fgfr2* knockout mice (referred here as cKO) on a mixed background have been previously described (13, 70). The conditional *Fgfr2* null allele harbors loxP recombination sites flanking regions encoding the Ig III binding and transmembrane domains of the *Fgfr2* gene (*Fgfr2f*) (71). Female mice homozygous for *Fgfr2f* alleles were crossed with male mice homozygous for *Fgfr2f* alleles and heterozygous for Cre recombinase transgene under human GFAP (hGFAP) promoter control, which is expressed in all dorsal telencephalic radial glia and astrocytes starting from embryonic day (E) 13.5 (72, 73). Cre negative offspring (referred to here as WT), littermates when possible, were used as control animals. Due to limited resources, only male offspring are used in this study because our previous study found ADHD-relevant behaviors in males (15). Childhood psychiatric disorders, particularly ADHD, have higher prevalence in males which allows for these data in males, while limited, to be translationally informative (74).

### 2.2 Behavioral procedures

Behavioral assessments were performed during the light cycle following at least 30 minutes of habituation prior to testing. Tests were always performed in the same order, as follows, from least to most stressful, as previously (15). In each cohort, mice came from two or more different litters to control for litter effects.

#### 2.2.1 Open field

In a chamber (20x14 inches), mice were tested for locomotor activity for 30 minutes each day for two days. Test mice were placed in a chamber corner, and movements were recorded using an overhead camera (AnyMaze videotracking software; Stoelting Co, Wood Dale, Illinois). To reduce effects of novelty seeking and/or anxiety, second day movement velocity and time spent in the center was measured to assess activity level and impulsivity respectively.

#### 2.2.2 Y-maze

In a Y-maze with three arms: A, B, and C (20 cm long, 10 cm wide, 20 cm high, separated by 120°), mice were tested for working memory by being placed in the distal end of arm A and allowed to explore for five minutes. Each individual arm entry (all four paws within arm) was recorded by an experimenter. The sequence of entries into the arms was broken into sets of three consecutive entries, each separated by one entry in the sequence. Spontaneous alternation, defined as the number of sets in which three different arms were entered divided by the total number of possible alternations, was used as the measure for working memory (75).

#### 2.2.3 Three-chamber social approach

In a three-chamber apparatus, mice were tested for social interaction. Prior to testing, each “stranger” mouse (WT animal of same sex, age, and strain as test mice from a separate cage) was habituated to an inverted wire cup within one side chamber for five minutes. Test mice were habituated to only the center chamber for 10 minutes. Then, the “stranger” mouse (social object) was placed within the inverted cup in one side chamber (counterbalanced across test mice), and an identical empty cup (non-social object) in the second side chamber. Test mice were recorded using an overhead camera (AnyMaze videotracking software; Stoelting Co, Wood Dale, Illinois) and evaluated for movement around the social vs. non-social objects. Social approach was quantified by comparing the time spent within 1 inch of the social object divided by time spent within 1 inch of the non-social object.

#### 2.2.4 Elevated plus maze

For anxiety-relevant behavior assessment, mice were placed at the junction of a maze with four arms, which were elevated from the ground, with two of them closed and two open, to explore the maze for 5 minutes. Movement was recorded using an overhead camera (AnyMaze videotracking software; Stoelting Co, Wood Dale, Illinois) and evaluated for time spent in each arm and center of the maze.

### 2.3 Neuroanatomical measurements

#### 2.3.1 Immunohistochemistry

Two weeks after behavioral testing, animals were anesthetized and perfused (phosphate buffered saline (1x PBS), then 4% paraformaldehyde), and brain tissue was post-fixed, cryoprotected (20% sucrose in 1x PBS), embedded in OCT compound, and cryostat (Leica, CM1900, Bannockburn, Illinois) sectioned at 50 μm thickness. Free-floating sections of regions with dopaminergic cells and terminals in which DAT has been shown to be relevant to ADHD (76): the medial frontal cortex (mFC, including prelimbic cortex and anterior cingulate cortex), nucleus accumbens (NAc), ventral tegmentum area (VTA), and dorsal striatum were stained using standard immunostaining methods with rat anti-DAT primary (1:5000, Chemicon) and anti-rat secondary (1:500, Vector Laboratories) antibodies. For pilot PV+ cell studies, sections with cortex and dentate gyrus were stained using standard immunostaining methods with mouse anti-parvalbumin primary (1:4000, Sigma) and Alexa dye-conjugated anti-mouse secondary (1:500-1000, Molecular Probes) antibodies.

#### 2.3.2 Light microscopy and punctal assessment

Digital images were taken of the mFC, VTA, NAc and the striatum using an AxioCam attached to Axio Phot microscope and AxioVision software (Carl Zeiss). Two to three sections (every tenth consecutive section) per region were imaged and analyzed for each brain, bilaterally. The same camera exposure and microscope lighting settings were used each time. Then, DAT puncta were quantified in ImageJ software (National Institutes of Health) by an experimenter blind to the experimental condition of each brain. The DAT immunostaining density values calculated were averaged: first, across sections for a given region for a given brain, and then across the brains in each group for each region separately.

#### 2.3.3 Stereology

Using StereoInvestigator (Microbrightfield) software coupled to an epifluorescent Axiocam microscope (Zeiss), the density of PV+ cells in mPFC (2–3 sections), total cortex (8–10 sections), and dentate gyrus (4–6 sections) was assessed using standard neurostereological methods for population and volume measurement.

### 2.4 Statistical analyses

Statistical tests were performed with SPSS version 21 and GraphPad Prism 11. Graphs were made with GraphPad Prism 11. Data normality and variance was assessed for all behavioral and neurobiological data sets. Outliers were identified and removed using the modified Thompson tau test (α = 0.05). Outcomes on each behavioral test and for each region’s DAT density or PV+ cell density were normalized to non-stressed (NS), wildtype (WT) littermate controls. Analyses of variance (ANOVA) and ANOVA for repeated measures outcomes were used to assess average velocity and total center time on day 2 in the open field, spontaneous alternation in the Y-maze, social approach in the three-chamber social task, and closed arm time in the elevated plus maze. Pearson correlations were performed to examine associations between DAT density, PV+ cell density, and behavioral measures. Group differences are expressed as percent change relative to control group.

## 3 Results

### 3.1 Prenatal stress enhances hyperactivity of *Fgfr2* cKO males but does not affect other ADHD-relevant phenotypes

To investigate how Fgfr2 cKO and prenatal stress independently and interactively impacted ADHD-relevant behaviors, we performed rodent behavioral tasks that allow for study of modeled ADHD symptoms. Because individuals with ADHD often experience symptoms of hyperactivity, impulsivity, working memory deficits, anxiety, and social functioning challenges (77), we used four rodent behavioral tasks: open field (to assess locomotor activity and impulsivity-relevant behaviors), Y maze (to assess working memory), three chamber social approach (to assess social intrusiveness), and elevated plus maze (to assess anxiety-relevant behaviors).

#### 3.1.1 Hyperactivity

**Figure 2:**
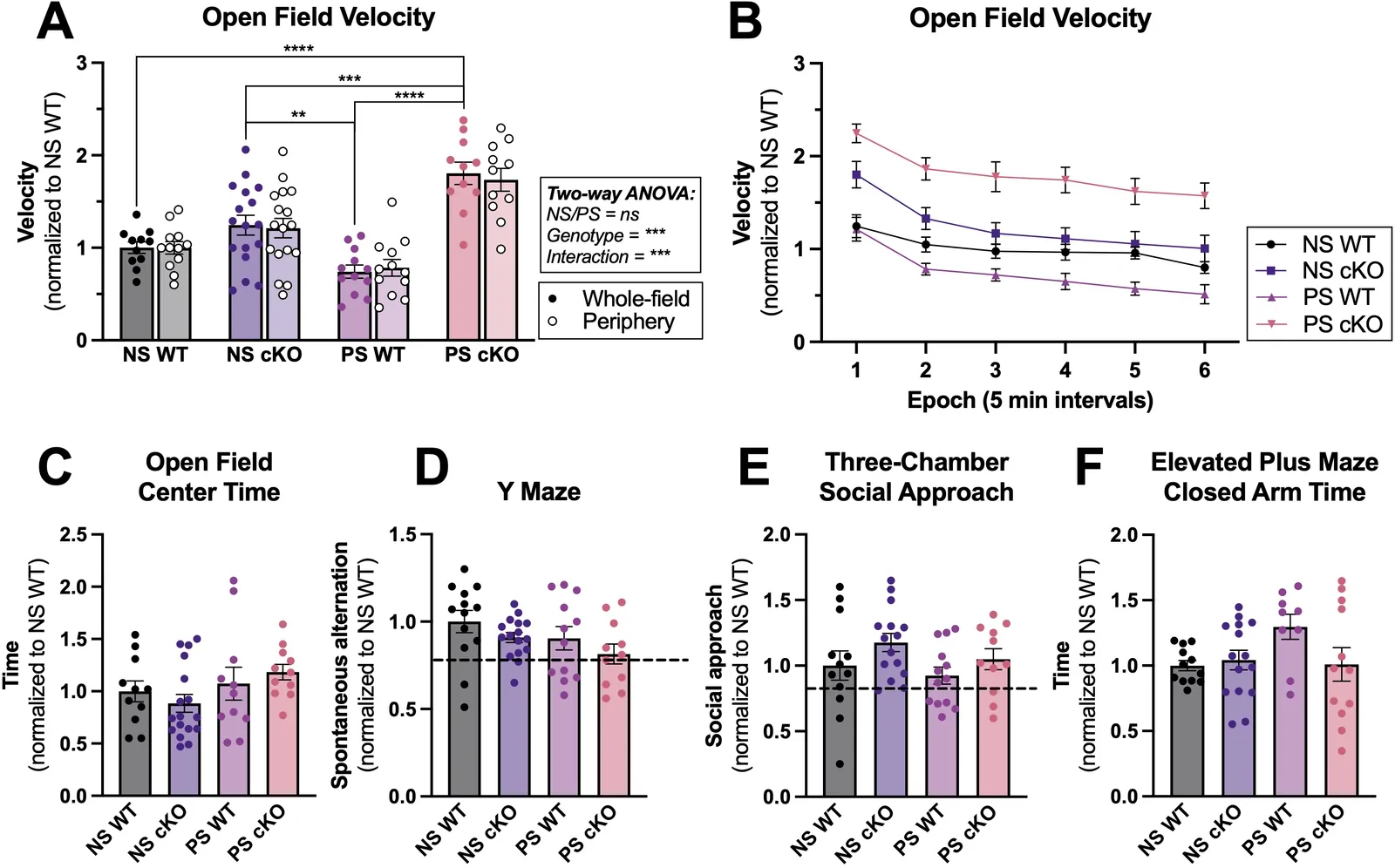
Prenatal stress enhances hyperactivity of *Fgfr2* cKO males but does not affect other ADHD-relevant phenotypes. All data are normalized to performance of the WT NS (control) group and presented as mean +/- standard error (SE). Two-way ANOVA results included in graphs: *** = p<0.001; ns = not significant. Bracketed lines indicate significant *post-hoc* comparisons after correcting for multiple comparisons: **** = p<0.0001, *** = p<0.001, ** = p<0.01. **(A)** Prenatal stress interacted with *Fgfr2* cKO to increase the whole-field velocity in the open field test, two-way ANOVA, *F*(1,47)=16.606, p <.0001. There was also a main effect of the Fgfr2 KO, *F*(1,47) = 42.576, p <.0001. n = 11 NS WT, 17 NS cKO, 12 PS WT, 11 PS cKO. **(B)** Across-session normalized velocity per 5-minute epoch across the 30min open field-testing session supported group differences in average whole-field velocity findings **(A)**. **(C)** Prenatal stress did not significantly affect impulsivity, measured as time spent in the center of the open field on the second day. There was a trend increase displayed by PS animals, *F*(1,46) = 2.990, p = 0.090. n = 11 NS WT, 17 NS cKO, 11 PS WT, 11 PS cKO mice. **(D)** Prenatal stress did not significantly impair working memory, as measured by spontaneous alternation in the Y-maze. The dotted line represents the normalized chance level of alternation at 0.78. Trend decreases were observed in spontaneous alternation in PS offspring, *F*(1,48) = 3.154, p = 0.081 and cKO animals, *F*(1,48) = 2.848, p = 0.098. n = 13 NS WT, 16 NS cKO, 12 PS WT, 11 PS cKO mice. **(E)** Social intrusiveness was not affected by prenatal stress, as measured by social approach in three chamber social task. The dotted line marks the normalized chance level of social approach at 0.83. The cKO groups displayed a trend increase in social approach, *F*(1,47) = 3.371, p = 0.073. n = 12 NS WT, 15 NS cKO, 13 PS WT, 11 PS cKO mice. **(F)** Prenatal stress did not significantly alter anxiety, measured as time spent in the closed arm of the elevated plus maze. There was a trend interaction between prenatal stress and *Fgfr2* cKO to increase anxiety in the PS WT group, *F*(1,43) = 3.076, p = 0.087. n = 12 NS WT, 15 NS cKO, 9 PS WT, 12 PS cKO.

The prenatally stressed *Fgfr2* cKO (PS cKO) group displayed the highest locomotor activity, as measured by average velocity in the open field (**Figure 2A**): a two-way ANOVA shows prenatal stress interacted with genotype to result in hyperactivity [*F*(1,47) = 16.606, *p* <.0001]. There was a 106% increase relative to the prenatally stressed wild-type (PS WT) offspring, and 80% increase relative to the non-stressed *Fgfr2* cKO (NS cKO) group. No significant change was observed in the PS WT group relative to NS offspring; PS did not exert a main effect [*F*(1,47) = 2.270, *p* = 0.139]. *Fgfr2* cKO alone increased locomotor activity/hyperactivity [*F*(1,47) = 42.576, *p* <.0001], consistent with our previous study (15). Across-session velocity was plotted (**Figure 2B**). All groups showed within-session habituation [*F*(5,47) = 40.690, *p* <.0001], but the absolute differences between groups remained the same; all interactions between time factor and other factors were nonsignificant. Finally, the average velocity across the whole field was compared to the less-aversive periphery, and no differences were observed in any of the groups (**Figure 2A**).

#### 3.1.2 Impulsivity

There were no statistically significant differences between groups in their impulsivity, which was measured as center time in the open field on the second day. There was, however, a trend increase in center time in PS offspring [*F*(1,46) = 2.990, *p* = 0.090]. PS groups spent on average 19% more time in the center than the non-stressed groups (**Figure 2C**). The directionality of this effect was in line with the extant literature (45, 46). The whole-field velocity was not lower from the periphery of the field (**Figure 2A**), confirming mice spent time in the center moving.

#### 3.1.3 Working memory

There were no significant differences in working memory performance as assessed by spontaneous alternation in the Y maze; there was only a trend decrease in performance in PS offspring [*F*(1,48) = 3.154, *p* = 0.082) and cKO offspring [*F*(1,48) = 2.848, *p* = 0.098] (**Figure 2D**). The interaction between the two factors was not significant [*F*(1,48) = 0.000, *p* = 0.995), suggesting trend summative deficits. All groups performed at or above chance level of spontaneous alternation.

#### 3.1.4 Social intrusiveness

Social intrusiveness, characterized by increased social approach in the three-chamber social task, was not displayed following prenatal stress [*F*(1,47) = 1.554, *p* = 0.219] (**Figure 2E**). There was a trend increase in social approach in cKO groups [*F*(1,47) = 3.371, *p* = 0.073]. The directionality of this effect consistent with the literature on *Fgfr2* cKO (15). All groups performed above chance level of social approach.

#### 3.1.5 Anxiety-relevant behavior

There were no main effects of prenatal stress [*F*(1,43) = 1.680, p = 0.202] or *Fgfr2* cKO [*F*(1,43) = 2.049, *p* = 0.160] on time spent in closed arms of the elevated plus maze. There was no significant interaction, only a trend of prenatal stress and genotype interacting [*F*(1,43) = 3.076, *p* = 0.087], such that anxiety-relevant behavior of PS WT animals was insignificantly increased compared to the rest of the groups. There was a 30% increase in closed arm time relative to PS cKO and NS WT groups. This pattern of effects, which is different from those observed for other measures, further suggests impulsivity and hyperactivity in open field was not confounded by anxiety.

### 3.2 DAT and PV+ cell density in medial prefrontal cortex

**Figure 3:**
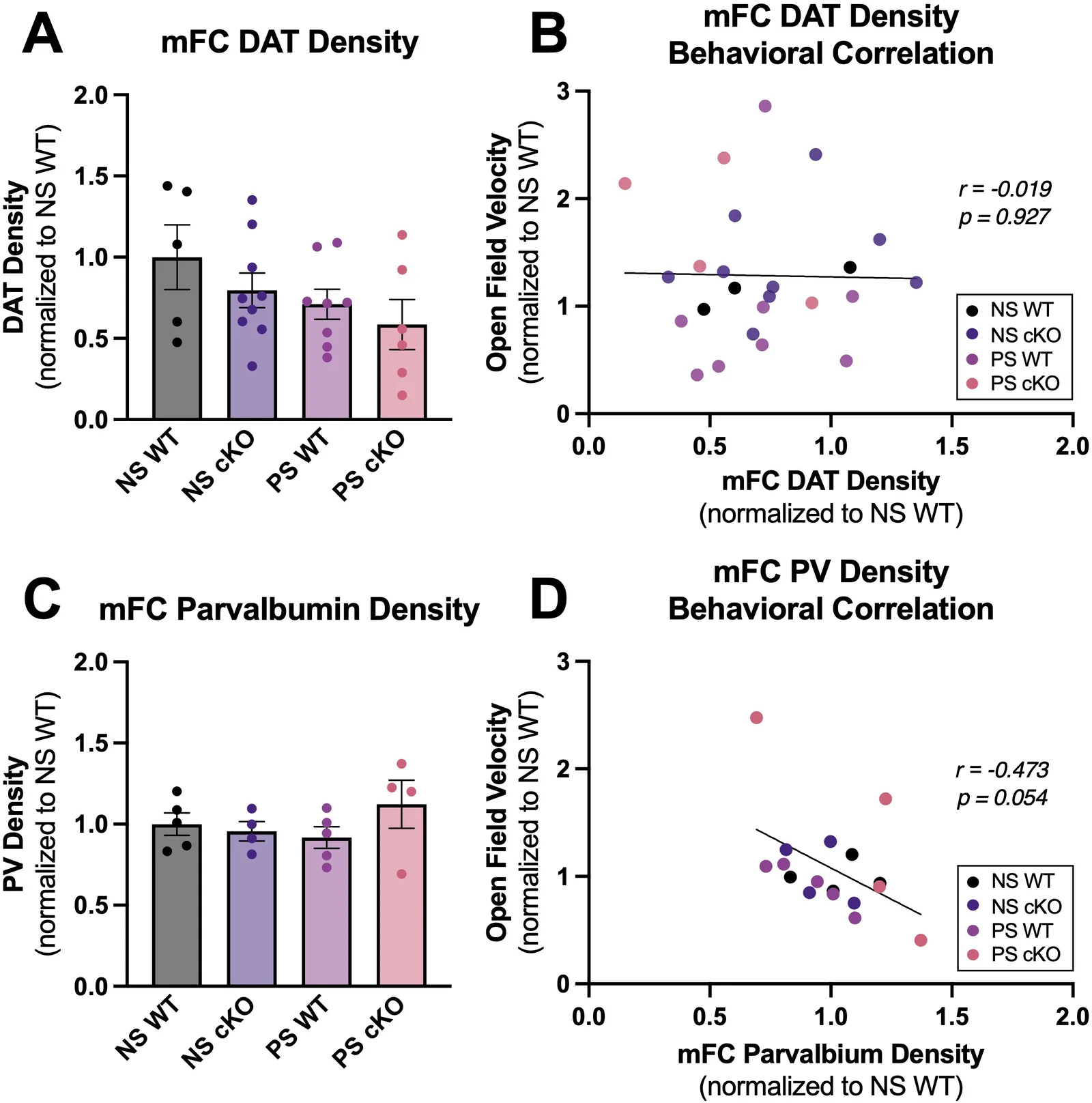
Prenatal stress did not significantly change DAT or PV+ density in the mFC. Data normalized to NS WT (control) group and presented as mean +/- SE, unless otherwise noted. **(A)** DAT density between groups in medial prefrontal cortex (mFC). Trend decrease displayed by PS groups in the mFC, *F*(1,24) = 3.540, p = 0.072. n = 5 NS WT, 9 NS cKO, 8 PS WT, 6 PS cKO. **(B)** There was not a significant correlation between open field activity and DAT density in the mFC, *r* = -0.019, n = 24, *p* = 0.927, two-tailed. **(C)** Pilot studies of parvalbumin (PV+) in mFC showed no differences between groups. n = 5 NS WT, 4 NS cKO, 5 PS WT, 4 PS cKO. **(D)** There was a trend correlation between open field activity and PV+ density in the mFC, *r* = -0.473, n = 17, *p =* 0.054, two-tailed.

To explore if abnormalities in cortical DA or inhibitory interneuron signaling occurred with behavioral outcomes, we measured dopamine transporter (DAT) protein and parvalbumin-positive (PV+) cell levels through immunohistochemistry (**Figure 3**). DAT uptakes DA into presynaptic terminals and is involved in temporal and spatial control of DA signaling (55) in medial frontal cortex, nucleus accumbens, and dorsal striatum. Its expression is tightly regulated (55, 78) and restricted to DAergic cells (79, 80). PV+ cells in dorsal forebrain (cortical and hippocampal regions) are fast-spiking interneurons for which the population density is carefully controlled because of their importance in excitatory-inhibitory balance and behavioral and cognitive regulation (81).

PS offspring did not have significantly different levels of DAT protein in any region. There was only a trend decrease with PS in DAT density in the medial frontal cortex (mFC) [*F*(1,24) = 3.540, *p* = 0.072], the projection area of DAergic neurons, exhibiting a 24% reduction relative to the NS animals (**Figure 3A**). There were no other effects in other regions (**Supplementary Figures 1A–E**). The pathway selectivity of these trend changes (e.g. primarily changes in the mesocorticolimbic as opposed to the nigrostriatal pathway) affected by prenatal stress is consistent with previous research (58, 62, 63). We then assessed potential correlations across individual animals between DAT density in regions affected by PS (mFC) and behaviors where PS offspring exhibited ADHD-relevant characteristics (hyperactivity) at trend or significant levels. In contrast to predictions, there were no significant or trend findings for any of the conducted correlations. To exemplify this: DAT density in the mFC plotted against open field activity is shown here (*r* = -0.019, n = 24, *p* = 0.927, two-tailed) (**Figure 3B**).

To begin exploring excitatory-inhibitory balance disruption, we performed a pilot study measuring PV+ cell density. There were no significant group differences in mFC (**Figure 3C**), though PV+ density showed trend correlations with open field activity (*r = -*0.473, n = 17, *p* = 0.054, two-tailed) (**Figure 3D**). Pilot results on PV+ cell density in other regions showed no group differences but a positive correlation of total cortex PV+ cell density with spontaneous alternation (**Supplementary Figures 1F–H**).

## 4 Discussion

Here, we performed a follow-up to our previous study (15) to explore potential prenatal gene-environment interactions with relevance to ADHD. Previously, we found both embryonic dorsal forebrain and early postnatal astroglial, but not adult, deletion of *Fgfr2* (*Fgfr2* cKO) led to locomotor hyperactivity (15) in males. In the current study, prenatal stress further increased locomotor hyperactivity but did not specifically alter other behavioral phenotypes of *Fgfr2* cKO male mice. This supports prenatal stress as a contributor to some part of ADHD-relevant outcomes, particularly in the presence of genetic vulnerabilities.

Several genome-wide scans and association studies (GWAS) have attempted to understand the genetic etiology of ADHD (82–85), with discovery of the first genome-wide significant risk loci in 2018 (84). Studies support the polygenic nature of ADHD, with evidence supporting polygenic risk scores have relevance across the longitudinal course of ADHD and co-occurring psychiatric conditions (86). Interestingly, genetic variations of *Fgfr2* have generally not been associated with ADHD in GWAS studies. One genome-wide quantitative trait locus (QTL) study of Korean children with ADHD identified four QTLs, which did include a QTL with *Fgfr2* as a potential candidate (87). Rather, disruptions in the fibroblast growth factor system are increasingly linked with other psychiatric conditions such as anxiety and depression (88–91). Nonetheless, examining *Fgfr2* cKO and other models may provide valuable insight into neurobiological mechanisms of ADHD-relevant behavioral alterations. In the current study, we assessed DAT levels across ADHD-relevant brain regions, as disruption of DA systems is highly implicated in ADHD and the target of the most effective treatments. DAT levels were not correlated with behavior, suggesting DAT dysregulation is not the mechanism of Fgfr2 cKO effects in our mouse model. However, human GWAS and other mouse transgenic models, including those manipulating FGF signaling, suggest multiple neuronal systems are involved in the pathophysiology of ADHD. These include disruptions in cortical interneurons (7, 11, 12, 65, 67), synaptic development (15, 92, 93) and norepinephrine signaling (94).

Prenatal stress has similarly been shown to affect such neuronal systems, including cortical and subcortical GABAergic cells: specifically, changes to migration of interneuron progenitors, and reduced inhibitory functioning in neonatal and adult forebrain have been shown (35). Prenatal stress has also been shown to impact development of dopaminergic systems (57, 95). The current study had only trend DAT alternations in medial prefrontal cortex (mFC), uncorrelated with behavior suggesting hyperactivity was underlaid by other brain changes. For example, though we had a small sample size in pilot studies of GABAergic cells (PV+ interneurons), our limited data thus far suggest PV+ density in mFC may be correlative with locomotor activity. Our pilot data are consistent with other evidence of cortical PV+ cell count correlating with hyperactivity (11).

Prenatal stress may also combine with genetic risks to affect epigenetic mechanisms, as emerging evidence points toward prenatal stress leading to DNA methylation changes (96). Epigenetic changes may partially reflect gene-environment interactions underlying ADHD etiology. DNA methylation changes have also been found in both periphery and brain in individuals with ADHD (97–100). The difficulty of studying gene-environment interactions of ADHD is further complicated by the spectrum of individual differences in clinical presentation, rearing environments, and polygenic risk scores. One interpretation of the results here is for ADHD, epidemiological studies of prenatal stress and GWAS studies may both fail to identify the contribution to risk if not accounting for effects from the other variable. It is challenging to account for the most important additional risk variables that may have interactive impacts on association studies. A study exploring the interaction between prenatal stress and polygenic risk scores for ADHD found prenatal stress may promote slower adolescent neurodevelopment in individuals with higher ADHD polygenic risk scores, but promote faster brain development in individuals with lower ADHD genetic vulnerability (101).

Gene-environment interactions are strongly implicated in the differential-susceptibility framework (102). While individual differences in responses to environmental factors have been well-appreciated, this framework counters the narrative that “vulnerable” individuals are disproportionately affected by *only* adverse experiences. Rather, individuals carrying different genetic markers may have differential susceptibility to *both* positive and negative experiences. In the current study, prenatally stressed WT animals displayed a trend increase in anxiety-relevant behavior, but prenatally stressed *Fgfr2* cKO mice did not. This finding may suggest potential protective factors from genetic “vulnerabilities” for certain behavioral phenotypes. This framework highlights opportunities for interventional strategies that include increasing positive experiences in modifying childhood psychiatric disease risk. Rodent studies of neurodevelopmental disorders models show enriched environments ameliorate behavioral abnormalities of transgenic mice (103, 104). These findings from enriched environment studies may be particularly relevant to ADHD. Evidence suggests children with genetic variants of dopamine receptor D4 [an identified vulnerability factor to ADHD (105)] are not only more adversely affected by poorer quality parenting, but they also benefit more from higher quality environments (102, 105). While positive rearing environment models have not yet been tested in *Fgfr2* cKO mice and no postnatal manipulations were done in the current study, early postnatal environmental conditions have been shown to induce persistent changes in offspring across the lifespan (106). Thus, early environmental conditions will be an important future direction to understand how they modify risk from ADHD-relevant genetic vulnerability and gestational stressors.

Overall, we found prenatal stress enhanced hyperactivity, but not other ADHD-relevant behaviors, in males with genetic vulnerability from a *Fgfr2 cKO* mouse model. Our findings may suggest environmental stressors exert selective effects on behaviors, with their impact depending on both genetic context and behavioral domain. Importantly, we cannot support whether effects are similar across sexes, as a limitation in our study was the exclusion of females. There has been evidence to suggest ADHD male bias may, in part, be due to underdiagnosis in females. This may be because females present with modified symptoms than males (107). Thus, future work should investigate both sexes and consider expanding behavioral tests. Due to the complexity of brain disorders such as ADHD, future work to uncover these complex gene-environment interactions will require transdisciplinary approaches integrating basic, clinic, and population-level research (108). Ultimately, improving our understanding of environmental modifiers of genetic risks will be beneficial for designing intervention strategies to minimize the burden of these disorders across the lifespan.

## Data Availability

The raw data supporting the conclusions of this article will be made available by the authors, without undue reservation.
