# Supplementary material for: Prenatal stress selectively enhances locomotor hyperactivity in male mice with embryonic loss of Fgfr2
Source: Front Psychiatry. 2026 Sep 3;17:1820942. doi: 10.3389/fpsyt.2026.1820942 (PMC13584961; doi:10.3389/fpsyt.2026.1820942)
Supplement: Supplementary file 1 [file DataSheet1.pdf]

SUPPLEMENTARY MATERIALS

Prenatal stress selectively enhances locomotor hyperactivity in male mice with embryonic loss of *Fgfr2*

Chen et al.

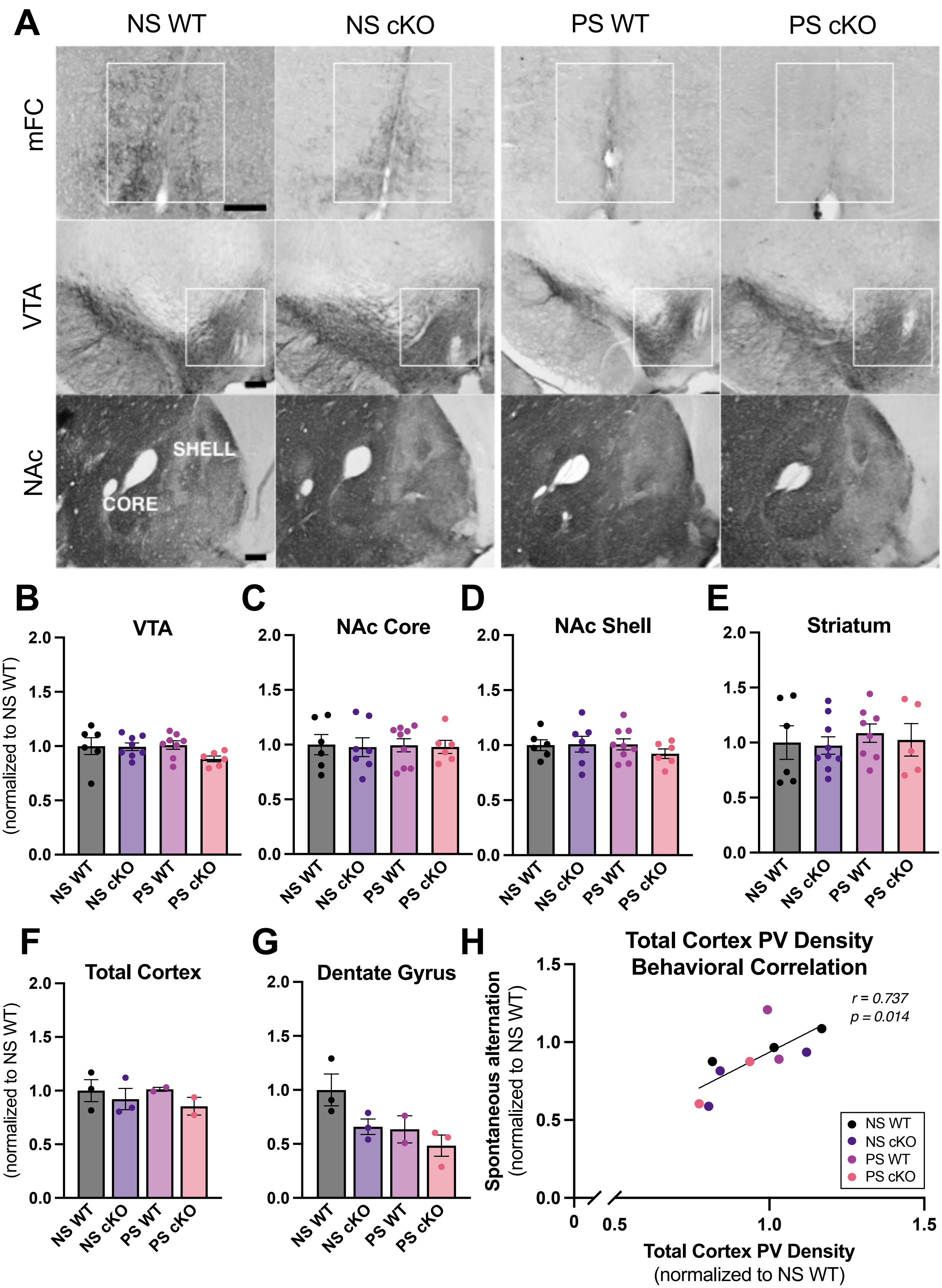
A
NS WT
NS cKO
PS WT
PS cKO
mFC
VTA
NAc
SHELL
CORE
B
VTA
DAT Density
(normalized to NS WT)
2.0
1.5
1.0
0.5
0.0
NS WT
NS cKO
PS WT
PS cKO
C
NAc Core
D
NAc Shell
E
Striatum
F
Total Cortex
PV Density
(normalized to NS WT)
G
Dentate Gyrus
H
Total Cortex PV Density
Behavioral Correlation
r = 0.737
p = 0.014
Spontaneous alternation
(normalized to NS WT)
Total Cortex PV Density
(normalized to NS WT)
0
0.5
1.0
1.5

Supplemental Figure 1: DAT density and PV+ density across brain regions. Data normalized to NS WT (control) group and presented as mean +/- standard error (SE). **A)** Representative images of staining for dopamine transporter (DAT; in dark) in the NS WT, NS cKO, PS WT and PS cKO groups, in the medial frontal cortex (mFC), ventral tegmental area (VTA), and nucleus accumbens (NAc), Scale bar is 100 μm. **B-E)** DAT density between groups across VTA, NAc core, NAc shell, and striatum. No trend or significant changes displayed by PS or cKO groups. n = 6 NS WT, 7-9 NS cKO, 8-9 PS WT, 5-6 PS cKO. **F-G)** Pilot studies of PV+ cell density in total cortex and dentate gyrus. In dentate gyrus, trend changes displayed by cKO groups, $F(1,7) = 4.468$, p = 0.0724 and by PS groups, $F(1,7) = 5.392$, p = 0.0532. No trend or significant changes in total cortex. n = 3 NS WT, 3 NS cKO, 2 PS WT, 2-3 PS cKO. **H)** There was a significant correlation between spontaneous alternation and PV+ density in the total cortex, $r = 0.737$, n = 10, $p = 0.014$, two-tailed.
